# The mechano-immune-vesicle regulatory circuit: a systems framework for bone homeostasis and regeneration

**DOI:** 10.1016/j.bioactmat.2026.05.050

**Published:** 2026-05-29

**Authors:** Ting Yang, Zhili Dong, Lili Chen, Xiaoxing Kou

**Affiliations:** a Hospital of Stomatology, Guanghua School of Stomatology, Guangdong Provincial Key Laboratory of Stomatology, Sun Yat-Sen University, Guangzhou, 510055, People's Republic of China; b South China Center of Craniofacial Stem Cell Research, Guanghua School of Stomatology, Sun Yat-Sen University, Guangzhou, 510055, People's Republic of China

**Keywords:** Bone remodeling, Mechanical forces, Immune response, Extracellular vesicles, Bone diseases

## Abstract

Bone remodeling is a mechanically adaptive process that integrates physical loading with immune regulation during homeostasis, repair, and disease. However, the traditional view fails to fully explain how mechanical and immune signals are coordinated across cellular compartments. Emerging evidence indicates that mechanical forces, immune responses, and extracellular vesicles (EVs) function as an integrated communication system rather than independent regulators. Here, we propose a systems-level framework termed the “mechano-immune-vesicle regulatory circuit”. In this framework, biophysical cues regulate EV biogenesis and selective cargo sorting through mechanotransduction pathways. These mechanically primed EVs then serve as communication vectors that reprogram osteoimmune responses, specifically by directing macrophage polarization, adaptive immunity, and bone-resident cell differentiation. The resulting immune output feeds back to reshape EV signaling and mechanosensitivity, suggesting a closed regulatory circuit that governs bone remodeling. By synthesizing advances in mechanobiology, osteoimmunology and EV biology, this review reframes bone remodeling as a mechano-immune-vesicle regulatory circuit rather than as a collection of parallel pathways. We further discuss how this framework may guide the design of mechano-responsive biomaterials and engineered EV-based therapies with spatiotemporal control over inflammation and bone regeneration. This conceptual integration provides a mechanistic basis for understanding bone diseases and for developing next-generation regenerative strategies.

## Highlights

• A systems-level mechano-immune-vesicle regulatory framework for bone homeostasis and regeneration is established.
• The mechanisms of mechanotransduction-driven EV cargo encoding and reciprocal osteoimmune crosstalk are elucidated.
• Strategies for designing mechano-responsive biomaterials with spatiotemporal immunomodulatory control are highlighted.
• Translational challenges and future directions for engineered EV-based regenerative therapies are explored.

## Graphical abstract

**Figure:**
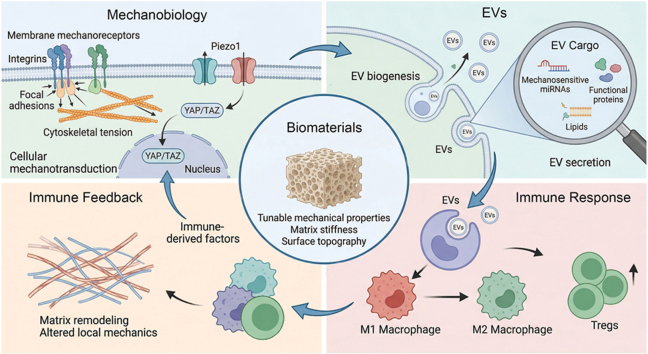

## 1 Introduction

Bone remodeling is a complex and continuous physiological process essential for maintaining skeletal integrity, repairing microdamage, adapting to mechanical demands, and regulating systemic mineral homeostasis throughout life [1]. This process depends on the balanced removal of old bone matrix by bone-resorbing osteoclasts and the deposition of new bone tissue by bone-forming osteoblasts [2]. These activities are spatially and temporally coupled within transient structures known as bone multicellular units (BMUs), thereby preserving bone mass and structural integrity are preserved under normal conditions [3]. Historically, bone remodeling was thought to be regulated primarily by systemic hormones and local cytokines [4]. However, accumulating evidence has revealed a more intricate regulatory network, in which the interplay among mechanical forces, the immune system, and EV-mediated intercellular communication jointly govern skeletal health and disease [[5], [6], [7]]. Accordingly, this review synthesizes recent advances in bone remodeling, with a focus on its regulation by mechanical forces, the immune responses, and EV-driven intercellular communication.

Mechanical stimuli regulate key cellular processes, including gene expression, protein synthesis, proliferation, and differentiation [[8], [9], [10]]. Wolff's law, a cornerstone of skeletal biology, states that repetitive bone loading triggers adaptive responses that enhance mechanical resilience [11]. This adaptation relies on mechanotransduction, through which cells convert mechanical cues into biochemical signals [12]. Osteocytes act as primary mechanosensors and master regulators of bone remodeling, coordinating osteoblast and osteoclast activity to direct bone formation or resorption [[13], [14], [15], [16]]. However, the molecular and cellular mechanisms by which this system orchestrates remodeling remain incompletely understood.

The recognition that immune cells critically regulate bone remodeling, including responses around bone biomaterials, has given rise the field of “osteoimmunology”, which investigates bidirectional interactions between the skeletal and immune systems within the bone marrow [17,18]. Immune cells orchestrate bone remodeling by driving post-fracture inflammation and modulating bone cell differentiation and activity, while their crosstalk with bone cells underlies both physiological bone turnover and pathological bone loss [[19], [20], [21], [22], [23]]. Although the underlying mechanisms remain incompletely defined, the functional coupling between the immune system and bone remodeling is now widely recognized as a central principle in skeletal biology.

EVs, once considered merely cellular “garbage disposal” systems, are now recognized as key intercellular messengers that deliver proteins, lipids, and nucleic acids to modulate recipient cell function [24,25]. They provide an important link between mechanical signaling and osteoimmune responses because mechanical stress modulates EV secretion and function, positioning EVs as major effectors of mechanotransduction [[26], [27], [28]]. EVs released by bone cells can recruit or polarize immune cells, whereas immune cell-derived EVs reciprocally regulate bone cell differentiation and activity to sustain bone homeostasis [[29], [30], [31]].

Accordingly, we define the mechano-immune-vesicle regulatory circuit as a closed-loop regulatory framework in which mechanical cues from the bone microenvironment are sensed by resident cells, encoded into EV-mediated biochemical signals, and subsequently transmitted to immune cells to regulate osteoimmune homeostasis. The immune responses elicited by these vesicular signals can, in turn, remodel the extracellular matrix, alter osteogenic and osteoclastic activity, and reshape the local mechanical niche, thereby feeding back to the initial mechanical input. Thus, this circuit is defined by directional signal conversion, vesicle-mediated intercellular communication, and immune-driven mechanical feedback, rather than by a simple coexistence of mechanical, immune, and vesicular events.

## 2 Regulation of bone remodeling by mechanical forces

The skeleton is a dynamic organ that continuously adapts to physical forces and this adaptation is a fundamental determinant of its mass, geometry, and structural integrity. The principle that bone structure is shaped by its mechanical loading represents a core concept of skeletal biology [32]. The conversion of mechanical stimuli (e.g., compression, tension, fluid shear stress, and matrix stiffness) into a coordinated cellular and biochemical responses is known as mechanotransduction [33]. Mechanotransduction signaling is essential for maintaining physiological bone homeostasis and serves as the primary driver of adaptive bone remodeling, as exemplified by orthodontic tooth movement (OTM) [34]. In this context, mechanical cues should be viewed not only as physical inputs but also as upstream regulators that determine how bone-resident and immune cells generate soluble mediators, EV-associated signals, and remodeling outcomes. Emerging evidence indicates that this process is far more complex than a simple cellular reaction, involving the integration of mechanosensing, metabolic shifts, neural input, and critically, the modulation of the local osteoimmune microenvironment [35,36].

### 2.1 Mechanosensing and intracellular transduction pathways

Osteocytes, comprising over 90% of bone cells, are strategically embedded within the mineralized matrix and form an extensively interconnected network through their dendritic processes [37]. This unique anatomical positioning makes them the primary mechanosensors of the skeleton [38], enabling them to perceive mechanical strain and the associated interstitial fluid flow within the lacuno-canalicular system [39]. Representative mechanosensors including Piezo-type mechanosensitive ion channel component 1 (Piezo1), transient receptor potential vanilloid subfamily 4 (TRPV4), integrins, and force-activated surface proteins collectively form a multilayered apparatus, together with the pericellular matrix, primary cilium, and caveolae. These structures couple mechanical strain to intracellular signaling by tethering cells to the ECM, buffering membrane tension, and organizing signaling complexes [[40], [41], [42], [43], [44], [45]]. Mechanosensitive microRNAs such as miR-365 and miR-146a are similarly regulated by mechanical stress in chondrocytes and osteoblasts, linking force sensing to inflammatory activation [46]. The dentoalveolar complex provides a particularly informative in vivo model, in which periodontal ligament stem cells (PDLSCs) and alveolar bone marrow mesenchymal stem cells (aBMSCs) sense and transduce mechanical forces through TRPV4 and CD109 to regulate their phenotype and the balance between osteogenesis and osteoclastogenesis. In this setting, Piezo1 appears dispensable for force-induced remodeling, whereas the kinin B2 receptor pathway acts as a negative regulator of the remodeling cascade [[47], [48], [49], [50], [51], [52]]. Thus, distinct mechanical cues are first discriminated at the sensor level before being converted into downstream remodeling signals.

Once perceived, physical signals are transduced through intracellular hubs that link the cytoskeleton to the nucleus and initiate metabolic reprogramming. Key pathways include the Wnt/β-catenin signaling, a potent osteogenic driver that upregulates osteogenic marker genes expression such as *Runx2* and *Sp7* [53]. Notably, mechanical loading reduces the expression of sclerostin, a Wnt signaling antagonist secreted by osteocytes, thereby facilitating osteogenesis [54]. Furthermore, the transcriptional regulators Yes-associated protein (YAP) and transcriptional co-activator with PDZ-binding motif (TAZ) have emerged as dominant force-activated sensors that spatially couple osteoblast precursor mobilization and angiogenesis to the mechanoregulation of osteogenesis [28,55]. Upon activation by force, YAP translocates to the nucleus and forms a complex with the NF-κB p65 subunit, a master regulator of inflammation and osteoclastogenesis [56]. This YAP/NF-κB p65 axis directly links mechanical stimulation to the sterile inflammatory cascade required for bone resorption [57]. Concurrently, osteoblastic signal transducer and activator of transcription 3 (STAT3) activity is closely associated with exercise-induced bone mass and orthodontic force-driven alveolar bone remodeling [58,59]. Mechanical force can also physically modulate protein dynamics to maintain homeostasis. For instance, physiological stress promotes receptor-independent endocytosis of TNF-α, which then binds to mTORC2 to restrain mTOR signaling and preserve MSC stemness [60]. In addition, cyclic mechanical stretch induces profound metabolic shifts: aBMSCs respond to force by increasing lactate production, which is then secreted as a paracrine signal to modulate adjacent cells [47]. These pathways therefore function as a cue-to-output relay, translating force magnitude, duration, and tissue context into transcriptional, metabolic, and secretory programs.

This entire cascade of local cellular events, ranging from ion flux and protein activation to metabolic and epigenetic shifts, ultimately converges on the production of downstream effector molecules that orchestrate remodeling activity [61]. The resulting signaling is further refined at the post-transcriptional level by mechanosensitive miRNAs that regulate the expression of key osteogenic and osteoclastic factors [62]. These primary signals induce inflammatory cytokines (IL-1β, IL-6, TNF-α) and chemokines, thereby creating a localized, sterile inflammatory microenvironment [63]. Notably, force-induced inflammation, which involves specific programmed cell death pathways such as caspase-1-dependent pyroptosis, constitutes an essential adaptive mechanism rather than a merely pathological side effect [64,65]. These inflammatory mediators, together with matricellular proteins such as osteopontin (OPN) and growth factors such as BMPs, recruit and activate effector cells, including CCR2^+^ macrophages and osteoclasts, to execute the bone remodeling process [66,67]. Beyond local remodeling, mechanical loading also exerts systemic effects by prompting osteocytes to release miR-196a-5p-enriched EVs that target brown adipose tissue, thereby promoting thermogenesis and combating obesity [68]. Together, these findings indicate that mechanical forces are biologically encoded into both soluble and vesicular signals, providing the upstream basis for EV-mediated osteoimmune communication. This complex integration is further modulated by the peripheral nervous system [69] and can be systemically disrupted, as seen in type 1 diabetes mellitus where mechanotransduction is blunted and remodeling responsiveness is impaired [70].

### 2.2 Mechanical forces shaping the osteoimmune microenvironment

Mechanical force is not merely a local cue for bone cells but also a powerful environmental signal that shapes the osteoimmune microenvironment by modulating immune cell composition and function, a process enabled by the intrinsic mechanosensitivity of immune cells [71,72]. Monocytes, macrophages, T cells, and B cells use their antigen receptors (TCRs, BCRs) as refined mechanosensors to probe the physical properties of their surroundings, and this force-dependent receptor-cytoskeleton coupling is fundamental to immune activation, viral invasion, and host defense [[73], [74], [75], [76], [77], [78]]. B lymphocytes, which are central to osteoimmunology through their production of RANKL and OPG, are likewise regulated by mechanical cues that influence their activation and differentiation [79]. Macrophages and monocytes are especially responsive to physical forces that govern key functions such as phagocytosis; Piezo1 acts as a core mechanosensitive ion channel that translates cyclical forces into innate immune programs, promotes anti-inflammatory phenotypes, and can drive macrophage proliferation through the Piezo1-AKT-Cyclin D1 axis [[80], [81], [82], [83]]. Other forces, including fluid shear stress, also regulate immune activation and can be exploited in pathology. In osteoarthritis (OA), for example, Piezo1-controlled glycolysis in MSCs promotes Th17 recruitment and activity, whereas mechanical loading within an inflamed joint can disrupt Treg feedback and perpetuate chronic inflammation. [[84], [85], [86]]. These examples position immune cells as active decoders of tissue mechanics rather than passive responders to bone-cell-derived signals.

**Fig. 1:**
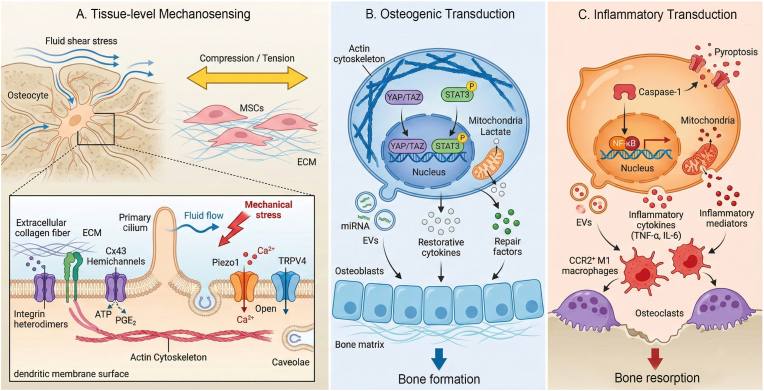
Mechanotransduction pathways regulating bone homeostasis. (A) Mechanical stimuli, including fluid shear stress, compression and tension are sensed by membrane receptors including integrin β1, TRPV4 and Piezo1, triggering Ca^2+^ influx. (B) Within physiological ranges, mechanical stress promotes actin filament reorganization, nuclear translocation of YAP/TAZ and p-STAT3, and mitochondrial lactate production. These intracellular shifts promote the release of EVs and restorative factors that polarize macrophages toward a regenerative M2 phenotype to support osteoblast-mediated bone formation. (C) Excessive or dysregulated stress triggers a pro-inflammatory cascade involving caspase-1-mediated pyroptosis and NF-κB signaling. This leads to the secretion of inflammatory cytokines that skew macrophages toward an M1 phenotype, thereby favoring bone resorption.

Within bone, this mechano-immune crosstalk is a central mechanism driving adaptive remodeling [87]. In OTM, applied mechanical force triggers a sterile inflammatory response that is indispensable for remodeling, marked by CCR2^+^ macrophage recruitment to the pressure side, where these cells promote osteoclastogenesis [64,88]. Multi-omics analyses have begun to map the underlying network, revealing chemokine gradients produced by mechanosensitive periodontal ligament cells (PDLCs) that guide macrophage trafficking [89]. Beyond chemokines, force-induced mitochondrial transfer from macrophages to BMSCs has emerged as a novel mechanism that directly enhances stem cell osteogenic capacity [90]. Together, these findings show that mechanical inputs are converted into immune-cell recruitment, polarization, metabolic exchange, and bone-cell differentiation, thereby providing a mechanistic rationale for mechanical interventions as therapeutic strategies (Fig. 1).

## 3 The mechano-immune-vesicle regulatory circuit

The traditional view of bone remodeling often treats mechanical forces, immune cells, and EVs as parallel regulators. However, emerging evidence supports a paradigm shift: these components do not function in isolation but converge into an integrated crosstalk network that coordinates the bone remodeling [91]. Here, we propose the mechano-immune-vesicle regulatory circuit as a dynamic, closed-loop feedback system in which biophysical cues, vesicular transport, immune responses and bone microenvironment remodeling are functionally interconnected. In this network, mechanical forces provide the biophysical input, EVs serve as relevant communication vectors, and the immune system functions as the central signal processor that feeds back to remodel the bone microenvironment. Interactions involving only mechanical regulation of bone cells, immune regulation without EV participation, or EV-mediated communication without evidence of mechanical responsiveness or feedback remodeling are discussed as related mechanisms, but are not considered complete examples of this circuit. Elucidating the bidirectional signaling pathways within this system, specifically how one factor initiates a cascade that in turn modulates the others, is crucial for uncovering pathological mechanisms and developing novel therapeutic strategies for bone regeneration.

### 3.1 Mechanical loading dictates EV biogenesis and cargo sorting

A foundational axis of this network is the translation of physical stimuli into molecular signals, a process in which EVs act as important mediators. EVs are heterogeneous population of lipid bilayer-enclosed particles that are broadly categorized by biogenesis and size into exosomes (30-150 nm), microvesicles (100-1000 nm), and apoptotic vesicles (1-5 μm) [[92], [93], [94], [95]]. Acting as both regulators and carriers, EVs convey information linking the mechanical environment, local immune status, and metabolic activity of bone-resident cells [96,97]. Mechanical stimuli, including fluid shear stress, compression, tension and ECM stiffness can directly regulate EV biogenesis, cargo loading, and biological efficacy, thereby modulating their therapeutic potency [98,99]. For example, applying continuous normal stress to the surface of MSCs via centrifugal rotation platforms increases EV secretion yield more than 4-fold, while substantially enhancing their inherent regenerative bioactivity [100]. Similarly, culturing macrophages in a mechanically stimulated bioreactor that combines static topography with dynamic fluid stimulation significantly increases their EV yield [26]. The cellular mechanical microenvironment not only alters yield but also reshapes the secretory phenotype; for instance, optimizing substrate mechanics for MSCs produces EVs with markedly superior therapeutic efficacy in complex tissue repair settings [101]. Furthermore, mechanosensitive EV biogenesis facilitates inter-tissue communication across the broader musculoskeletal system. For instance, cyclic mechanical strain stimulates myotubes to release miR-92a-3p-enriched EVs, which inhibit osteoclastogenesis by targeting the PTEN/AKT pathway in osteoblasts [102]. Similarly, fluid shear stress stimulates muscle cells to release miR-196a-5p/miR-155-5p-enriched EVs, which suppress osteoclastogenesis by inhibiting mitochondrial biogenesis in osteoclast precursors [103]. Additionally, EVs derived from cyclic stretch-induced PDLCs transfer miR-181d-5p to osteoblasts, inhibiting the TNF signaling pathway and promoting osteogenic differentiation [104].

Cells sense these physical cues through multiple mechanotransduction pathways. A central mechanism involves mechanosensitive ion channels, particularly Piezo1, which translate physical forces into biochemical signals [105]. Piezo1 activation triggers extracellular Ca^2+^ influx, which in turn promotes the nuclear translocation of the transcriptional co-activator YAP [106]. For example, dynamic mechanical loading, including fluid flow and stretching, enhances YAP activity and upregulates vesicle-trafficking proteins, thereby markedly boosting EV production [107]. When mechanical stretch reaches a critical threshold, mechanically induced nonlinear Ca^2+^ elevation activates the scramblase anoctamin-6, initiating EV-mediated membrane cholesterol efflux and implicating EVs as active effectors of cellular mechanical adaptation [108]. ECM stiffness, which imposes tension on cells, is also a powerful modulator of EV production. Fibroblasts cultured on substrates with different stiffness exhibit significant changes in EV size and composition, an effect abolished by inhibition of mechanotransduction [109]. Mechanistically, matrix stiffness and mechanical pressure activate focal adhesion kinase (FAK) via integrins, driving pronounced F-actin rearrangement into stress fibers [110]. This reorganized actin network not only provides physical tracks for multivesicular bodies (MVBs) to dock and fuse with the plasma membrane, thereby accelerating EV release; the resulting localized cytoskeletal tension also acts as a molecular sieve. Specifically, this tension selectively sorts miRNAs, notably miR-21-5p, miR-143-3p, and miR-125b-5p, as well as essential cargo-loading proteins, into the secreted EVs [[111], [112], [113]]. In addition, mechanical stress such as fluid shear stress directly reprograms intracellular vesicle trafficking, profoundly amplifying EV release through the simultaneous upregulation of the MVB-docking proteins Rab27a/b and downregulation of the lysosome-directed protein Rab7 [114,115]. Fluid shear stress also activates specialized metabolic mechanotransducers in osteocytes to produce small EVs through Ank-mediated pyrophosphate regulatory pathways [116]. Finally, extreme mechanical stimuli such as acute shear stress can induce a compensatory survival pathway by promoting autophagosomes-MVB, which rescues intracellular autophagy components from lysosomal degradation and redirects them for release via EVs [117].

### 3.2 Mechanically primed EVs drive osteoimmune reprogramming

Once released, EVs function primarily as immunomodulators within the network. EV-mediated immunomodulation is a cornerstone of the osteoimmune axis because it regulates the local inflammatory microenvironment required for successful tissue regeneration [118]. Although mechanical forces and immune cells can communicate through direct cell-cell contact and soluble cytokines, these mechanisms are constrained by spatial proximity and enzymatic degradation. EVs provide a distinctive link in this triad because their unique properties help overcome these barriers [119]. Their lipid bilayer protects fragile cargoes from degradation in the extracellular space, while their nanoscale size enables them to seamlessly navigate the lacuno-canalicular network and support more distant, targeted communication [120,121]. Importantly, this long-range transport is actively modulated by ECM biomechanics which affect both EV biogenesis and spatial distribution [122]. Upon reaching target cells, EVs initiate multifaceted communication either by binding to specific cell-surface receptors that trigger downstream signaling cascades or by entering cells through internalization mechanisms [123]. These mechanisms include direct fusion of the EV membrane with the target-cell plasma membrane and vesicle internalization through clathrin- or caveolin-mediated endocytosis [124]. Ultimately, this molecular transfer alters recipient-cell phenotypes, including proliferation, differentiation, and migration, thereby governing tissue homeostasis and disease pathogenesis [[125], [126], [127]].

Substantial evidence highlights MSC-derived EVs (MSC-EVs) as important mechanosensitive regulators of the local immune landscape. [128]. The therapeutic potential of MSCs, skeletal stem cells (SSCs), and human umbilical cord mesenchymal stem cells (hUCMSCs) is related to the ability of their EVs to polarize pro-inflammatory macrophages (M1 macrophages) toward anti-inflammatory macrophages (M2 macrophages) [[129], [130], [131]]. Specifically, BMSC-EVs promote tendon-bone healing by delivering miR-23a-3p to drive M1-to-M2 macrophage polarization, a shift that supports the anabolic phase of tissue regeneration [132,133]. In addition, MSC-EVs exert substantial control over adaptive immunity by destabilizing the Th17 transcription factor RORγt, thereby correcting the local Treg/Th17 imbalance and limiting osteoimmune pathology [134,135]. The physical microenvironment further dictates this immunomodulatory capacity. Altering cellular substrate stiffness directly modulates the MSC-EV secretory phenotype, yielding vesicles with enhanced M2-polarizing capability [101]. This biological activity depends on the specific cargo packaged during EV biogenesis. Cyclic mechanical stretch directly primes adipose-derived stromal cells (ADSCs) to package regenerative miRNAs, notably miR-877, into EVs that counteract the pro-inflammatory niche to support coupled angiogenesis and osteogenesis [136]. MSC-EVs also alleviate neuroinflammation by delivering cargo that inhibits the NLRP3 inflammasome, thereby reducing pro-inflammatory cytokine production and pyroptotic cell death [137]. This finding provides a direct mechanistic link by which EVs suppress inflammatory pyroptosis, a key driver of pathological bone loss.

Bone-resident cells, including osteocytes, chondrocytes, fibroblasts, and PDLCs, serve as local mechanosensors that use EVs to orchestrate osteoimmune crosstalk. For instance, mechanically strained osteocytes selectively package miR-3110-5p and miR-3058-3p into EVs to promote osteoblastic differentiation [138]. The trajectory of this immune regulation depends on the mechanical thresholds. Under physiological cyclic stretch, PDLCs secrete EVs that inhibit NF-κB signaling in macrophages, suppress IL-1β production and maintain periodontal homeostasis [139]. Conversely, under orthodontic remodeling loads, PDLC-EVs become enriched with miR-9-5p, which targets SIRT1 to activate NF-κB and induce the sterile inflammation required for tooth movement [140]. This mechanically induced inflammatory niche is further amplified by periodontal fibroblasts, which release mitochondrial DNA (mtDNA)-enriched EVs in response to compressive orthodontic forces. After internalization by local macrophages, this EV-associated mtDNA activates the NLRP3 inflammasome and triggers macrophage pyroptosis, contributing to orthodontic alveolar bone remodeling [141].

Finally, immune cell-derived EVs feedback to steer the bone microenvironment toward either destruction or regeneration [142,143]. Because macrophages play a major role in bone homeostasis, their EVs can mediate of osteoimmune interactions [144,145]. The function of these EVs aligns with the polarization state of the parent macrophage; M1-derived EVs (M1-EVs) generally promote inflammation, whereas M2-derived EVs (M2-EVs) exert anti-inflammatory and pro-regenerative effects [146]. In periodontitis models, M2-EVs mitigate inflammation and bone loss by reprogramming the local immune milieu, for example by inducing an Anxa1(hi) neutrophil subpopulation that enhances osteogenesis and angiogenesis [147]. During OTM, M2-EVs accelerate bone formation by activating the MeCP2-TCF20-HDAC1 axis in PDLSCs [148]. Similarly, macrophage-EVs influence cementoblast mineralization through Wnt and BMP signaling to preserve root integrity during force application [149]. The cargo of these immune EVs is mechanically regulated. For instance, applying fluid shear stress to M2 macrophages mechanically downregulates miR-423-5p packaging, thereby promoting BMSC osteogenesis by relieving Suv39h1 repression [150] (Table 1). This immunomodulatory function is closely linked to cell metabolism; for example, EV-releasing hydrogels can promote diabetic bone reconstruction by inducing macrophage metabolic reprogramming, highlighting the translational potential of mechanically responsive EVs for regenerative therapy [151,152].

**Table 1:** Regulatory effects of mechanically primed EVs on the osteoimmune microenvironment.

Cell source	Mechanical condition	Target pathway	Key cargo molecules	Effects on osteoimmune microenvironment	Reference
BMSCs	Not mentioned	M2 macrophage polarization	miR-23a-3p	Promote tendon-bone healing	[132]
ADSCs	Mechanical stretch	Counteract the pro-inflammatory niche	miR-877	Restore osteoimmune balance to rescue osteogenesis	[136]
Osteocytes	Mechanical strain	Osteoblastic differentiation pathways	miR-3110-5p, miR-3058-3p	Promote osteoblastic differentiation	[138]
PDLCs	Cyclic stretch force	Inhibit NF-κB pathway to reduce IL-1β	Not specified	Maintain periodontal immune homeostasis	[139]
PDLCs	Cyclic stretch force	Target SIRT1 to activate NF-κB	miR-9-5p	Induce sterile inflammation required for OTM	[140]
Fibroblasts	Orthodontic force	Activate the NLRP3 inflammasome	mtDNA	Trigger macrophage pyroptosis required for OTM	[141]
M2 Macrophages	Orthodontic force	MeCP2-TCF20-HDAC1	Not specified	Promote orthodontic bone remodeling	[148]
M2 Macrophages	Fluid shear stress	Relieve Suv39h1 repression	Downregulation of miR-423-5p	Promote BMSC osteogenesis	[150]

### 3.3 Immune feedback on mechanosensitivity and matrix mechanics

The loop is closed when EV-modulated immune responses feedback to reshape extracellular matrix organization, mineralization, bone resorption, and local stiffness, thereby redefining the mechanical cues sensed by bone-resident cells. At the receptor level, pro-inflammatory cytokines such as IL-1β and TNF-α directly remodel the mechanosensory apparatus of bone-resident cells. Notably, IL-1α upregulates Piezo1 expression in chondrocytes through the MAPK-p38-CREB signaling pathway, thereby increasing cellular deformation under mechanical load [[153], [154], [155]]. This creates a pathogenic positive-feedback loop, particularly in OA-associated cartilage degeneration, where inflammation amplifies Piezo1 sensitivity. Consequently, otherwise physiological loads can trigger further tissue damage, which in turn perpetuates chronic inflammation [156,157].

Concurrently, these inflammatory cytokines reprogram the EV secretory machinery of stromal cells, including fibroblast-like synoviocytes. Exposure to TNF-α or IFN-γ alters both the quantity and cargo composition of released EVs [158,159]. Rather than promoting repair, this immune-primed environment enriches pathological miRNAs, such as miR-31a-5p and miR-16, within EVs, thereby driving osteoclastogenesis and cellular apoptosis [160,161]. Thus, although mechanical stimulation can intrinsically increase secretion of osteogenic or immunomodulatory EVs, the concurrent immune context determines whether the resulting vesicular output is regenerative or destructive [162,163].

Finally, effector immune cells and their secreted EVs complete this circuit by directly reshaping the physical architecture of the ECM. During the transition from inflammation to tissue repair, macrophages regulate the secretion of matrix metalloproteinases (MMPs) and collagenases, and orchestrate collagen cross-linking through enzymes such as lysyl oxidase (LOX) [164,165]. This immune-driven ECM assembly alters the viscoelasticity, fiber alignment, and local stiffness of the bone microenvironment [166]. Such immune-mediated changes in ECM stiffness feed back into the FAK-YAP/TAZ signaling axes within resident bone cells [167]. Consequently, they reset their baseline mechanosensitivity and influence subsequent cellular responses to mechanical load or injury [41]. In three-dimensional osteogenic niches, macrophage-driven modifications of matrix stiffness can effectively override baseline material properties to dictate the osteogenic fate of co-cultured MSCs [168]. Ultimately, this bidirectional reciprocity, in which immune-modified matrix mechanics continuously reset cellular mechanotransduction, is a key mechanism governing both skeletal homeostasis and disease progression [169].

**Fig. 2:**
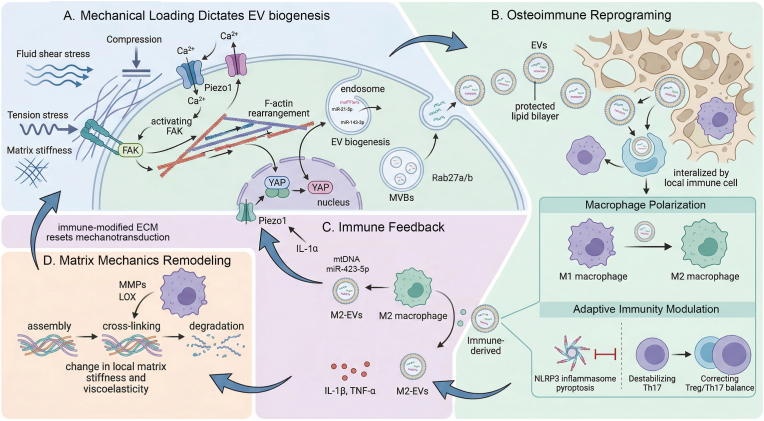
Mechanotransduction and osteoimmune crosstalk via EVs. (A) Mechanical stimuli, including fluid shear stress, tension, and matrix stiffness, are sensed by Piezo1 and FAK, triggering Ca^2+^ influx and F-actin rearrangement. This mechanotransduction activates the YAP signaling pathway to drive endosomal EV biogenesis. (B) Osteoimmune reprogramming occurs when these EVs are internalized by local immune cells, promoting M2 macrophage polarization, restoring the Treg/Th17 balance, and inhibiting NLRP3-mediated pyroptosis. (C) Immune feedback is established through M2-EVs and immune-derived factors, such as IL-1α, which upregulate Piezo1 expression to modulate cellular mechanosensitivity. (D) Matrix mechanical remodeling is driven by macrophage-secreted MMPs and LOX, which regulate ECM assembly and cross-linking. These alteration in local stiffness and viscoelasticity reset the mechanotransduction environment, creating a reciprocal regulatory loop between mechanical forces and the immune niche.

In summary, the convergence of mechanobiology, immunology, and EV research has revealed a sophisticated regulatory network that governs bone remodeling. Mechanistically, external biophysical forces are first biologically encoded into EV biogenesis and selective cargo sorting of EVs. These mechanically primed EVs subsequently act as precise communication vectors that modulate the phenotypic polarization of local immune cells and the differentiation of bone-resident cells. The circuit is then completed by the reciprocal immune feedback: the resulting immune output may reshape the subsequent EV biogenesis in parent cells and reset baseline mechanosensitivity. Future investigations aimed at dissecting this closed-loop crosstalk and leveraging its components, including mechano-responsive biomaterials and engineered EVs, may hold substantial promise for improving the management of bone health and disease (Fig. 2).

## 4 Pathological dysregulation of the mechano-immune-vesicle circuit

In pathological states, the delicate balance of the mechano-immune-vesicle circuit is compromised, and disruption of any single link, including abnormal mechanical loading, aberrant EV secretion, or immune dysregulation, can precipitate a vicious cycle of skeletal degeneration. This pathological uncoupling typically begins with aberrant physical forces that drastically reprogram EV biogenesis and cargo. On one hand, abnormal overloading directly provokes joint-resident cells to secrete pathogenic EVs. In OA, excessive mechanical stress induces subchondral osteocytes to increase EV secretion, thereby mediating destructive cross-tissue communication. Specifically, EV-delivered miR-23b-3p targets OTUD4 to disrupt mitophagy in articular cartilage chondrocytes, thereby accelerating OA progression [170]. Similarly, in temporomandibular joint (TMJ) OA, aberrant biomechanical loading, such as unilateral anterior crossbite, upregulates EV biogenesis and secretion from condylar chondrocytes and drives abnormal cartilage calcification. Consistently, local injection of EVs obtained from chondrocytes stimulated by fluid flow shear stress into the TMJs of normal rats induced cartilage degeneration and calcification [171]. On the other hand, mechanical unloading, as occurs in disuse osteoporosis, disrupts homeostasis by reducing ApoV production through Piezo1 downregulation and reduced of Ca^2+^ influx [172]. Additionally, unloading actively promotes bone loss by selectively packaging miR-92b-3p into EVs that suppress osteogenesis through ELK4 inhibition, thereby preventing osteoblast precursors from differentiating into mature osteoblasts. [173]. Mechanical forces also regulate pathological EV uptake. For instance, low fluid shear stress upregulates the expression levels of adhesion molecules, such as MCAM and PECAM-1, thereby increasing the uptake efficiency of EVs carrying pathological signals [174]. Subsequently, these mechanically altered EVs act as primary instigators of cellular dysfunction and abnormal immune responses. Using primary chondrocytes and rat models of TMJ and knee OA, recent work from our laboratory revealed that under inflammatory biomechanical stress, IL-1β-induced hybrid EVs can physically trigger irreversible mitochondrial structural damage. This organelle-specific assault subsequently leads to a critical depletion of TCA cycle metabolites (specifically acetyl-CoA and α-KG), thereby driving chondrocyte senescence via metabolic reprogramming [175].

**Fig. 3:**
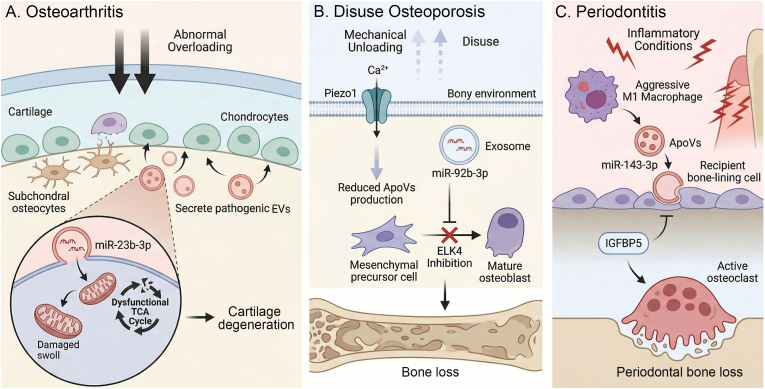
Dysregulation of the mechano-immune-vesicle axis in bone diseases. (A) Osteoarthritis: abnormal mechanical overloading triggers subchondral osteocytes to secrete pathogenic EVs containing miR-23b-3p. These EVs are internalized by chondrocytes, leading to mitochondrial damage and a dysfunctional TCA cycle, which ultimately drives cartilage degeneration. (B) Disuse Osteoporosis: mechanical unloading and disuse inhibit Piezo1-mediated Ca^2+^ influx in bone environment, resulting in reduced ApoV production. Simultaneously, exosomal miR-92b-3p targets ELK4, inhibiting the differentiation of mesenchymal precursor cells into mature osteoblasts and leading to progressive bone loss. (C) Periodontitis: under inflammatory conditions, aggressive M1 macrophages release ApoVs enriched with miR-143-3p. These vesicles are taken up by recipient bone-lining cells, where they suppress IGFBP5 and promote osteoclast activation. This cascade accelerates pathological bone resorption, resulting in significant periodontal bone loss.

Finally, this aberrant osteoimmune microenvironment feeds back to further distort EV properties, thereby consolidating the pathological loop. For instance, in inflammatory conditions such as periodontitis, M1 macrophage-derived ApoVs exacerbate periodontal bone resorption by delivering miR-143-3p, which directly inhibits the osteogenic factor IGFBP5 in recipient cells, uncouples bone remodeling and promotes bone loss [176]. This pathological milieu alters not only the biochemical cargo of EVs but also their intrinsic biophysical traits. Inflammatory conditions and abnormal mechanical forces can synergistically alter EV membrane morphology, softness, and nanomechanical deformability [177]. These structural shifts directly govern the EV mechanical resistance, lubricating property, pathological uptake efficiency, and subsequent drug delivery potential within damaged joints or periodontal tissues [178,179]. Because these complex biophysical cues ultimately redirect stem cell fate, targeting such mechano-immunologically modulated EVs has emerged as a highly promising therapeutic strategy for skeletal and periodontal diseases [180] (Fig. 3).

## 5 Applications and translational prospects

Elucidating the integrated mechano-immune-vesicle network provides a powerful conceptual framework for designing the next-generation bone regeneration therapies. By deciphering the convergence of mechanical, immune, and EV-mediated signaling, researchers can move beyond single-target approaches toward multifaceted strategies that actively guide endogenous healing process [181]. Although targeting this network holds considerable promise, clinical translation faces major challenges related to safety, efficacy, and accessibility. Overcoming these hurdles will require continuous innovation and integration of emerging technologies. Ultimately, the future of bone regeneration lies in highly personalized and adaptive therapeutic systems.

### 5.1 Therapeutic strategies and temporal regulation

To effectively harness the mechano-immune-vesicle regulatory circuit for bone regeneration, traditional cell-free therapies must overcome hurdles of rapid local clearance and insufficient target specificity [182,183]. A primary strategy is to optimize EV cargo and scale up manufacturing before administration, particularly by exploiting mechanotransduction pathways [184]. Because conventional static cultures produce low EV yields, modern biomanufacturing uses dynamic mechanical stimuli to increase production while enriching pro-regenerative cargo. For example, controlled fluid shear stress applied to parent cells via vertical-wheel or seesaw-motion bioreactors significantly boosts both EV secretion and therapeutic potency [185,186]. This mechano-biomanufacturing paradigm is further advanced by microfluidic platforms, physiologically relevant laminar-flow systems, and audible acoustic waves that yield surface stress, all of which can precisely tune physical cues to optimize vesicle biofabrication [[187], [188], [189]]. Alongside mechanical priming, pretreating MSCs with the stress modulator TUDCA enhances both the production and the osteo-immunomodulatory potency of their EVs, leading to superior bone regeneration [190]. Beyond priming, direct EV modification enables the creation of targeted vesicles that modulate immune crosstalk. For instance, fusion of M2 macrophage-EVs with BMSC-EVs creates chimeric nanovesicles that effectively modulate macrophage polarization and accelerate early bone formation [191]. Furthermore, clinical interventions can synergize with the mechanical environment; for example, cell microparticles loaded with pro-inflammatory factors and chloroquine can cooperate with applied mechanical forces to facilitate OTM by transiently amplifying beneficial inflammation [192].

Rather than acting as passive delivery vehicles, modern biomaterial scaffolds are designed as active biophysical modulators of the mechano-immune-vesicle network [193]. A fundamental design principle is to mimic of the native mechanical environment and guide endogenous mechanosensing. By precisely tuning scaffold stiffness, viscoelasticity, and hierarchical topography via 3D-printing, materials can provide bone-mimetic biomechanical stimuli that actively recruit and rejuvenate senescent autologous BMSCs [[194], [195], [196]]. This tailored mechanical environment also modulate host immunity to fuel the EV circuit. For example, specific biophysical and chemical cues from scaffolds (such as lithium-doped calcium silicate) can polarize macrophages toward an M2 phenotype, which subsequently secrete their own pro-regenerative, miR-145-5p-enriched EVs, effectively transforming the host's innate immune system into an autonomous EV factory at the defect site [197]. At the nanoscale, macrophages sense biomaterial elasticity via Piezo1, triggering a Ca^2+^/CAMK2A-RAB8 cascade that directly regulates EV secretion and cargo sorting [198]. By tuning these specific nanomechanical cues, next-generation biomaterials can precisely orchestrate the local osteoimmune environment for advanced tissue regeneration.

**Fig. 4:**
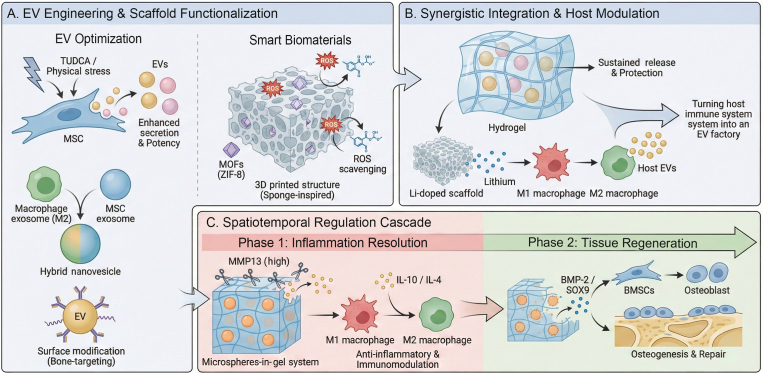
Integrated strategy for bone regeneration using engineered EVs and smart biomaterials. The schematic contrasts the traditional bone tissue engineering paradigm, which is limited by cell viability and immunogenicity, with a cell-free EV-based approach that faces challenges in EV yield and clearance. Current solutions combine EV engineering with smart biomaterial design. These components are integrated into an intelligent hydrogel system that enables synergistic spatiotemporal regulation: anti-inflammatory EVs are released first to resolve early-stage inflammation and polarize macrophages, followed by pro-regenerative EVs that drive late-stage vascularized bone formation, thereby recapitulating the natural healing cascade.

Finally, these approaches are converging on the paradigm of spatiotemporal regulation, which aims to recapitulate the dynamic, phased cascade of the natural mechano-immune-vesicle circuit. During early bone regeneration, a moderate inflammatory response is necessary to remove necrotic tissue, whereas later stages require an anti-inflammatory and pro-repair microenvironment for matrix mineralization [21]. Intelligent, ECM-mimicking hydrogel systems are being developed to bridge biophysical support with this temporal requirement. Logic-based hydrogel systems are developed to meet this temporal requirement. For instance, a logic-based microspheres-in-gel system first releases anti-inflammatory IL-10^+^ EVs in response to high MMP13 levels, which indicate the inflammatory phase, and then followed by a sustained release of pro-chondrogenic SOX9^+^ EVs to drive the repair phase [199]. Similar sequential-release strategies, such as resolving chronic inflammation before delivering engineered bacterial EVs loaded with osteogenic factors, demonstrate how integrating tunable biomechanical platforms and engineered EVs can be integrated to achieve programmed, spatiotemporally controlled regeneration [200,201]. This synergistic integration represents a key translational application of the mechano-immune-vesicle axis (Fig. 4).

### 5.2 Challenges in clinical translation and future technological directions

Translating the mechano-immune-vesicle regulatory circuit from bench to bedside requires overcoming hurdles that extend beyond generic EV therapeutics [202,203]. A primary challenge is the standardized biomanufacturing of highly specific, mechano-responsive EVs. Although bioreactors that dynamically mimic the physical microenvironment can enhance therapeutic cargo, standardizing these complex mechanical inputs to achieve Good Manufacturing Practice (GMP)-compliant, consistent, clinical-scale yields remains a major bottleneck [204,205]. Furthermore, conventional isolation methods are poorly suited to preserve delicate mechanically sorted cargoes, such as specific mechanosensitive miRNAs and lipids, that are required for precise immunomodulation, making quality control and potency assay standardization exceptionally difficult [[206], [207], [208]].

Post-manufacturing challenges include unpredictable in vivo behavior and delivery. Upon systemic administration, native EVs exhibit a short plasma half-life and non-specific biodistribution, which severely dilute their localized mechano-immunomodulatory effects at the defect site [209,210]. Bridging this gap requires advanced biomaterial-based delivery systems, such as EV-in-Hydrogel platforms. However, these systems introduce new complexities: EV degradation kinetics and spatiotemporal release must closely match the dynamic stages of tissue pathophysiology while providing appropriate baseline biophysical cues, such as stiffness, to synergistically guide local immune cells [211].

The clinical adoption of these sophisticated therapies is further constrained by the lack of specific regulatory frameworks for dynamically engineered EVs [212]. Overcoming these translational hurdles will increasingly demand the convergence of synthetic biology and artificial intelligence to design programmable EVs that can flawlessly integrate into and correct pathological mechano-immune networks [213,214].

In summary, deciphering the mechano-immune-vesicle network marks a new direction in regenerative medicine. By rationally designing biomaterials and EV-based therapies to modulate crosstalk among mechanical forces, immune cells, and EVs, the field may move beyond simple tissue replacement and toward true physiological regeneration.

## 6 Conclusion and prospects

The understanding of bone remodeling has progressed from a focus on isolated hormonal and cellular actions to recognizing it as a multifactorial, integrated network. This review synthesizes evidence suggesting the synergistic interplay among mechanical forces, the immune system, and EVs as a central axis coordinating skeletal homeostasis and repair. Mechanical stimuli provide architectural cues; the immune system acts as a dynamic sensor and director; and EVs serve as distinctive messengers that facilitate multicellular communication. These elements do not operate in parallel but are deeply interconnected: mechanical cues are translated into EV-based signals that shape the osteoimmune microenvironment, whereas immune cell activation dictates EV cargo, which in turn modulates bone cell fate. This continuous feedback loop enables spatiotemporal adaptation to physiological demands or injury, and its dysregulation underpins many skeletal pathologies.

This network-centric perspective marks an emerging paradigm in bone regenerative medicine, shifting the field from uniform interventions toward personalized, multitargeted strategies that precisely modulate the local mechano-immune-vesicle network and harness the innate regenerative potential. Translating this vision into therapy requires will require continued interdisciplinary collaboration. Despite persistent challenges, elucidating this complex crosstalk provides a clear roadmap. By learning to interpret and manipulate the cellular language of forces, cytokines, and vesicles, future therapies may restore not only skeletal architecture, but also its full physiological function.

## Ethics approval and consent to participate

Confirm that no ethical issues are involved.

## CRediT authorship contribution statement

**Ting Yang:** Conceptualization, Investigation, Visualization, Writing – original draft, Writing – review & editing. **Zhili Dong:** Conceptualization, Investigation, Visualization, Writing – original draft, Writing – review & editing. **Lili Chen:** Conceptualization, Project administration, Supervision, Writing – review & editing. **Xiaoxing Kou:** Conceptualization, Project administration, Supervision, Writing – review & editing.

## Declaration of competing interest

All authors declare no conflicts of interest.
